# Health impact assessment of air pollution in megacity of Tehran, Iran

**DOI:** 10.1186/1735-2746-9-28

**Published:** 2012-12-17

**Authors:** Kazem Naddafi, Mohammad Sadegh Hassanvand, Masud Yunesian, Fatemeh Momeniha, Ramin Nabizadeh, Sasan Faridi, Akbar Gholampour

**Affiliations:** 1 Department of Environmental Health Engineering, School of Public Health, Tehran University of Medical Sciences, Tehran, Iran; 2 Center for Air Pollution Research (CAPR), Institute for Environmental Research (IER), Tehran University of Medical Sciences, Tehran, Iran; 3 Center for Solid Waste Research (CSWR), Institute for Environmental Research (IER), Tehran University of Medical Sciences, Tehran, Iran

**Keywords:** Tehran air pollution, Air Q software, Health impact assessment, Mortality, Morbidity

## Abstract

The aims of the present study were to provide quantitative data on the impact of air pollution on the health of people living in Tehran city, the most populated city of Iran. The approach proposed by the World Health Organization (WHO) was applied using the AirQ 2.2.3 software developed by the WHO European Centre for Environment and Health, Bilthoven Division. Concentrations of ozone, nitrogen dioxide, sulfur dioxide and particulate matter of aerodynamic diameter ≤ 10 μm (PM_10_) were used to assess human exposure and health impacts in terms of attributable proportion of the health outcome, annual number of excess cases of mortality for all causes, and cardiovascular and respiratory diseases. The annual average of PM_10_, SO_2_, NO_2_ and O_3_ in Tehran were 90.58, 89.16, 85 and 68.82 μg/m^3^, respectively. Considering short-term effects, PM_10_ had the highest health impact on the 8,700,000 inhabitants of Tehran city, causing an excess of total mortality of 2194 out of 47284 in a year. Sulfur dioxide, nitrogen dioxide and ozone caused about, respectively, 1458, 1050 and 819 excess cases of total mortality. Results indicate that the magnitude of the health impact estimated for the city of Tehran underscores the need for urgent action to reduce the health burden of air pollution.

## Introduction

Exposure to air pollution can cause both acute (short-term) and chronic (long-term) health effects. The acute effects of air pollution on human health were amply proven in the 20th century, when severe air pollution in Europe (Meuse Valley and London) and in the United States (Donora, Pa) caused deaths and disease in hundreds of thousands of people [1,2]. This episode has demonstrated that high concentrations of air pollution lead to an increase in mortality [2] and morbidity. In later studies, investigators found that levels below even current air pollution guidelines are associated with adverse effects on health [3-13] and air quality standards may not be protective enough for the most sensitive groups [14]. In addition, even though pollution due to combustion fuels has fallen dramatically in recent years, emerging pollutants such as O_3_ and NO_X_, and changes in the composition and size distribution of particulate matter, have become important in the health effects of air pollution [3,15].

**Figure 1:**
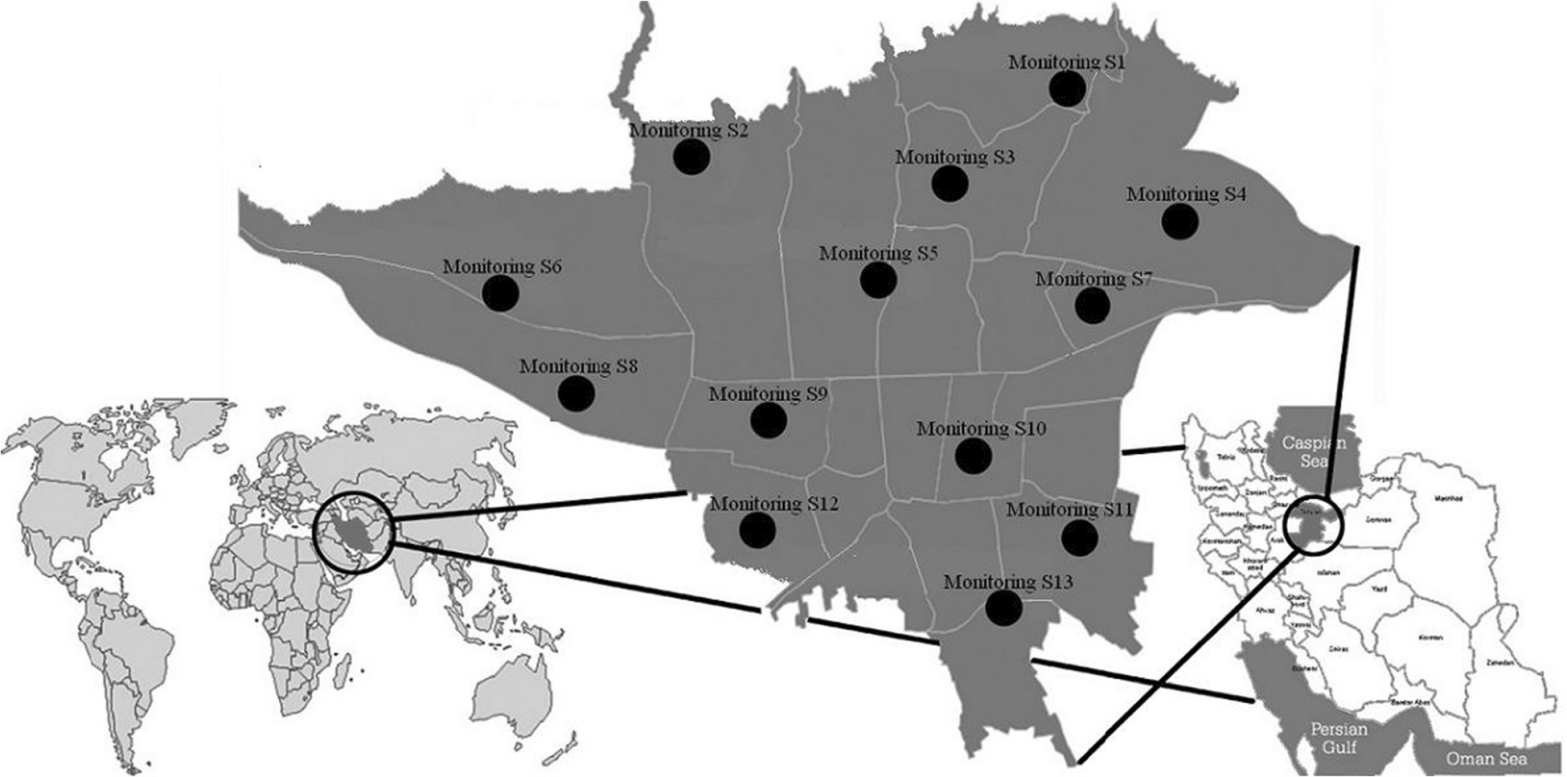
Map of the study area and locations of monitoring stations (Monitoring S) with valid observations.

The present study assessed the health impact of air quality on the residents of Tehran, the capital city of Iran, the largest urban area of Iran with a population of 8,700,000 in 2011 (Figure 1) [16]. The city also is ranked as one of the largest cities in Western Asia and 19th in the whole world. As in other large cities, Tehran is faced with serious air quality problems. In general, 20% of the total energy of the country is consumed in Tehran. Pollutants such as PM_10_, SO_2_, NO_2_, HC, O_3_ and CO are the major air pollutants in Tehran, about 80-85% of which is produced by mobile sources of pollution [8].

The city has a capacity for 700,000 registered cars yet 3 million roam its streets on a daily basis. Compounding Tehran’s air pollution problem is its geographical location. With the location of 35° 41' N - 51° 25' E and altitude of 1000–1800 meters above mean sea level, Tehran is located in valleys and is surrounded on the north, northwest, east and southeast by high to medium high (3800–1000 m) mountain ranges. The mountain range stops the flow of the humid wind to the main capital and prevents the polluted air from being carried away from the city. Thus, during winter, the lack of wind and cold air causes the polluted air to be trapped within the city.

These concomitant conditions make Tehran as one of the worst areas in the world for atmospheric pollution with many days exceeding air quality standards during each year [17]. Because of the air pollution in the Great Tehran area, morbidity, mortality and symptoms emerge. At the moment, the concentration of these pollutants is higher than the standard level most of the time which means that they have numerous effects on the health of Tehran citizens [8].

Up to now the human health endpoints of air pollution has not been quantified in Tehran. The main aim of this study was the quantification of the short-term health effect of air pollutants for people living in Tehran, by using World Health Organization (WHO) approach.

## Materials and methods

### AirQ software

The approach proposed by the WHO was adopted using the Air Quality Health Impact Assessment (AirQ 2.2.3) software developed by WHO European Centre for Environment Health, Bilthoven Division. This program is used to estimate the impact of exposure to specific atmospheric pollutants on the health of people living in a certain period and area. The assessment is based on the attributable proportion (AP), defined as the fraction of the health outcome in a certain population attributable to exposure to a given atmospheric pollutant, assuming a proven causal relation between exposure and health outcome and no major confounding effects in that association. The AP can be easily calculated by the following general formula [18]:

(1) $$\text{AP}=\frac{\Sigma \left\{\left[\text{RR}\left(c\right)-1\right]\times P\left(c\right)\right\}}{\Sigma \left[\text{RR}\left(c\right)\times P\left(c\right)\right]}$$

Where AP is the attributable proportion of the health outcome, RR is the relative risk (RR) for a given health outcome, in category “c” of exposure, obtained from the exposure–response functions derived from epidemiological studies and *P*(c) is the proportion of the population in category “c” of exposure.

If the baseline frequency of the health outcome in the population under investigation is known, the rate attributable to the exposure can be calculated as

(2) IE=I×AP

Where IE is the rate of the health outcome attributable to the exposure and *I* is the baseline frequency of the health outcome in the population under investigation.

Finally, knowing the size of the population, the number of cases attributable to the exposure can be estimated as follows:

(3) NE=IE×N

Where NE is the number of cases attributed to the exposure and *N* is the size of the population investigated.

RR gives the increase in the probability of the adverse effect associated with a given change in the exposure levels, and comes from time-series studies where day-to-day changes in air pollutants over long periods were related to daily mortality, hospital admissions and other public health indicators. RR values used in the present assessment are shown in Table 1. The RR values used for PM_10_ were summary estimates derived from a quantitative meta-analysis of peer-reviewed studies focused on European investigations [19], while for SO_2_ the RR implemented in the software and proposed as summary estimate in the WHO Air Quality Guidelines for Europe [20] was used. Finally, for O_3_ and NO_2_ the RR values came directly from published studies on short-term effects within the Air Pollution and Health: a European Approach (APHEA) project [21,22]. The baseline rates of all mortality for the calendar year 2010 were obtained from death certificates recorded at the Civil Registration Office of Tehran. For hospital admissions, the baseline rates that proposed by WHO were used.

**Table 1:** Relative risk (RR) with 95% confidence intervals (95% CI), and corresponding reference, implemented in AirQ 2.2.3 software and used for the health effect estimates

**Health endpoint**		**Incidence**^**a**^	**RR (95% CI) per 10 μg/m**^**3**^			
			**O**_**3**_	**NO**_**2**_	**PM**_**10**_	**SO**_**2**_
Mortality	Total ICD-9-cm^b^ <800	543.5	1.003 (1.002–1.005)^e^[21]	1.003 (1.002–1.004)^c^[22]	1.006 (1.004–1.008)^d^[19]	1.004 (1.003-1.0048) [23]
	Cardiovascular ICD-9-cm 390–459	231	1.005 (1.002–1.007)^e^[21]	1.004 (1.003–1.005)^c^[22]	1.009 (1.005–1.013)^d^[19]	1.008 (1.002-1.012) [24]
	Respiratory ICD-9-cm 460–519	48.4	1.013 (1.007–1.015)^e^[21]	-	1.013 (1.005–1.020)^d^[19]	1.01 (1.006-1.014) [24]
Morbidity	HA^f^ Respiratory Disease	1260	-	-	1.008 (1.0048-1.0112) [20]	-
	HA COPD^g^	101.4	-	1.0026 (1.0006-1.0044) [22]		1.0044 (1–1.011) [25]
	HA cardiovascular disease	436	-	-	1.009 (1.006-1.013) [26]	-
	Acute myocardial infarction	132	-	-	-	1.0064 (1.0026-1.0101) [27]

^a^ Crude rate per 100,000 inhabitants.

^b^ International Classification of Diseases, 9th Revision, Clinical Modification (ICD-9-cm).

^c^ 1 h average.

^d^ Daily average.

^e^ 8 h “moving average”.

^f^ Hospital admissions.

^g^ Chronic obstructive pulmonary disease.

### Exposure assessment

Air concentrations of the selected pollutants in the area of interest were measured by Tehran Air Quality Control Corporation (TAQCC), Iran, using 25 permanent monitoring stations (Figure 1). Finally, the air pollutants data from the permanent monitoring station of Tehran city from January 2010 to January 2011 year were used.

There are 25 stations for the measurement of air pollution in Tehran, but some of them, based on WHO Criteria for Air Quality Health Impact Assessment, had an invalid data for assessment. Finally, data from 7, 10, 11, 13 and 13 stations located in north, west, south and central parts of Tehran that were consistently active during the study period for, respectively, PM_10_, SO_2_, NO_2_ and O_3_, were used.

For all the pollutants, the parameters required by the software (annual and seasonal maximum and annual 98th percentiles) were obtained and concentrations were recorded to 10 μg/m^3^ categories, corresponding to equivalent exposure categories. For O_3,_ data were expressed as an 8 h “moving average”; for NO_2_, as a 1 h average concentrations and for SO_2_ and PM_10_ as daily averages.

Exposure was estimated considering the city of Tehran as single agglomerate, with a residential population 8,700,000 people. The software assumes that concentrations measured are representative of the average exposure of the people. For example, if on 5% of sampling days concentrations were between 10 and 20 μg/m^3^, it was assumed that people were exposed to the corresponding concentration for 5% of their time.

## Results

Table 2 shows the summary of the statistics of environmental data in Tehran.

**Table 2:** Summary of the concentrations of air pollutants, and meteorological variables, Tehran (2010–2011)

	**Mean (SD)**	**Minimum**	**P25**	**P50**	**P75**	**P98**	**Maximum**	**No. of station**	**No. of days**
PM_10_, Annual 24 h (μg/m^3^)	90.58 (41.99)	22.54	60.34	86.65	114.80	175.68	329.95	7	2438
SO_2_, Annual 24 h (μg/m^3^)	89.16 (29.27)	45.47	69.24	84.47	101.60	161.45	313.27	10	3172
NO_2_, Annual 24 h (μg/m^3^)	85 (24.183)	49.37	68.88	77.72	95.70	145.88	163.85	13	4115
O_3_, Annual 8 h (μg/m^3^)	68.82 (40.48)	10.88	35.32	59.75	99.28	158.60	187.16	11	3530
Temperature (°C)	19.68 (9.87)	−5	10.69	21.10	28.92	38.12	42		365
Relative humidity (%)	38.87 (16.45)	6	27.82	34.23	48.70	62.84	100		365

No. Station: number of monitoring stations with valid observations.

No. days: number of days with valid observations.

The highest PM_10_ concentration was detected in the summer period with maximum value of 329.95 μg/m^3^. For O_3_ the maximum 8 h average concentration was 187 μg/m^3^ detected as expected in summer, while the maximum NO_2_ and SO_2_ concentrations, respectively, 163.85 and 313.27 μg/m^3^ were detected in winter.

The obtained results show that annual average of PM_10_ in Tehran was 1.3 times the world’s average (71 μg/m^3^) [17] and 4.5 times the guideline values prescribed by the WHO [28]*.* Also the annual average of NO_2_ in Tehran was 2.1 times the guideline value prescribed by the WHO [28].

**Figure 2:**
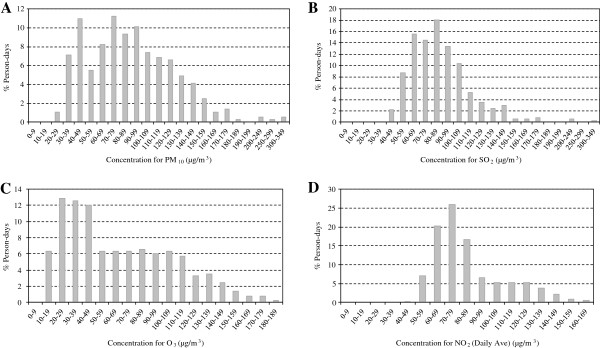
**Percentage of days on which people in Tehran are exposed to different concentrations of (A) PM**_**10**_**, (B) SO**_**2**_**, (C) NO**_**2 **_**and (D) O**_**3**_**.**

Figure 2A–D illustrates the concentrations of different pollutants and the percentage of time to which people were exposed to these concentrations. The data were used to estimate the short- term effects.

The short-term health impacts of exposure to PM_10_, SO_2_, NO_2_ and O_3_ above a reference value of 10 μg/m^3^ are shown in Table 3. The figures represent the estimated AP and number of excess cases in comparison to the cases if concentration values of the pollutants were 10 μg/m^3^. For O_3_, the numbers of excess cases over total mortality, cardiovascular and respiratory mortality (Table 3) were based on the RR values from the APHEA-2 project, which investigated health effects of ambient O_3_ in 23 European cities/areas for at least three years [21]. For NO_2_ the estimated short-term effects (Table 3) are based on the pooled estimates for the increase in mortality associated with an increase of 10 μg/m^3^ in 30 European cities participating in the APHEA-2 project [22].

**Table 3:** **Estimated attributable proportion (AP) expressed as percentage and number of excess cases in a year due to short-term exposure above 10 μg/m**^**3 **^**for PM**_**10**_**, SO**_**2**_**, O**_**3 **_**and NO**_**2**_

**Health Endpoints**	**Air pollutants**	**AP (uncertainty range)**^**a**^	**No. of excess cases (uncertainty range)**^**a**^
Total mortality	PM_10_	4.6 (3.14-6)	2194 (1486–2880)
	SO_2_	3 (2.33-3.67)	1458 (1102–1739)
	NO_2_	2.2 (1–2.93)	1050 (705–1389)
	O_3_	1.73 (1.16-2.85)	819 (549–1349)
Cardiovascular mortality	PM_10_	6.8 (3.89-9.35)	1367 (738–1916)
	SO_2_	5.98 (1.56-8.7)	1202 (315–1751)
	NO_2_	2.93 (2.22-3.64)	591 (446–733)
	O_3_	2.85 (1.16-3.95)	574 (233–794)
Respiratory mortality	PM_10_	9.53 (3.89-13.95)	402 (164–588)
	SO_2_	7.73 (4.55-10)	310 (192–422)
	O_3_	7 (3.4-8)	299 (143–341)
Hospital Admissions Cardiovascular Disease	PM_10_	6.8 (4.64-9.53)	2580 (1760–3617)
Hospital Admissions Respiratory Disease	PM_10_	6.09 (3.74-8.32)	6677 (4107–9126)
Hospital Admissions COPD	NO_2_	2.79 (0.3-6.64)	247 (27–586)
	O_3_	4.80 (2.52-7.09)	424 (222–626)
	SO_2_	3.38 (0–8.04)	298 (0–710)
Acute Myocardial Infarction	SO_2_	4.84 (2.02-7.43)	556 (233–854)
	NO_2_	2.65 (1.12-5.97)	305 (129–687)

^a^ Obtained using the lower and upper RR values.

## Discussion

This paper offers a study case using the WHO approach to assess the impact of atmospheric pollution on human health for people living in Tehran, one of the most populated areas in the world, where the geographical features make the air quality among the worst in the world. The impact was estimated as the increase in all-causes, cardiovascular and respiratory mortality, and hospital admissions for cardiovascular and respiratory diseases, COPD and acute myocardial Infarction for short-term exposure.

Considering short-term effects, PM_10_ had the greatest health impact on the 8,700,000 inhabitants of Tehran city, causing an excess of total mortality of 2,194 out of 47,284 people in a year. The effect of SO_2_, NO_2_ and O_3_ on total mortality was an excess of about, respectively, 1458, 1050 and 819 cases in a year.

The AirQ software has been used by other investigators to assess the human health impact of PM_2.5_[15,29] or PM_10_[30]. [15] estimated the human health risk in relation to air quality in two municipalities in an industrialized area of Northern Italy, the authors found that PM_2.5_ had the highest health impact on the 24,000 inhabitants of the two small towns, causing an excess of total mortality of 8 out of 177 in a year; also Ozone and nitrogen dioxide each caused about three excess cases of total mortality. [30] focused on short-term effects of PM_10_ in Trieste (about 200,000 inhabitants), a city in north-east Italy; For PM_10_ concentrations above 20 μg/m^3^, 52, 28 and 6 cases in excess, respectively, were estimated for total, cardiovascular and respiratory mortality. These figures, if normalized to the population in Tehran (8,700,000 inhabitants), would result in a number of excess cases very similar to those reported for PM_10_ in Table 2.

In another study, [31] found in Milan (1,308,000 inhabitants), a big industrial city in the Po Valley, the central estimate of the number of excess cases attributable to PM_10_ was 677 for total mortality. In a study of PM_10_ and O_3_ impact on human health in 13 Italian cities, with about nine million inhabitants during the period 2002–2004, [32] it was reported that on average 8220 deaths a year, excluding accidental causes, were attributable to PM_10_ above 20 μg/m^3^. For O_3_ the effect was estimated at 516 extra deaths yearly. For short-term effects exposure to PM_10_ above 20 μg/m^3^ was responsible for 1372 extra deaths.

Results obtained from studies of health effects of air pollution in various parts of the world differ. Particulate matter is the pollutant with the biggest health effects in all of these papers, including the present study.

This study has several limitations. One of the limitations of this approach is that the health impact focuses on individual compounds without considering the simultaneous exposure to several, which is what actually occurs [15]. The health effects of atmospheric pollution are indeed the consequence of interactions between different air contaminants, and between these and other compounds of natural origin. Generally, in quantitative assessments of health effects, the interactions between different contaminants are not investigated as it would require a good knowledge of the mechanisms of toxicity for the different compounds, which is rarely available. In order to take account of co-exposure to different pollutants, it is often assumed that the health effects of individual compounds are additive. However the simple addition of the effects of the single pollutants would not be right because atmospheric pollutants are usually positively related.

A further limitation related to the exposure assessment is that the approach assumes that concentrations measured in specific sampling points are representative of the average exposure suffered from people living in Tehran. Another limitation is due to the RR estimates derived in studies of different populations in comparison to the one under investigation. In particular, while for PM_10_, SO_2_, NO_2_ and O_3_ the RRs were mainly based on studies on the European population. The transferability of the mortality effect estimates from the evidentiary (e.g., the US cohort) to the target population, however, is feasible if no compelling evidence that the target population and the evidentiary population differ in the response to the air pollution [33].

## Conclusion

In conclusion, this study applied the AirQ software and the approach proposed by the WHO to provide quantitative data on the impact of PM_10_, SO_2_, NO_2_ and O_3_ on the health of people living in a certain area. The results are in line with those of other investigations and, despite the limitations, which are in common to similar studies, indicate that this method offers an effective and easy tool, helpful in decision-making.

The magnitude of the health impact estimated for the city of Tehran underscores the need for urgent action to reduce the health burden of air pollution. Local authorities, through policies that aim mainly to reduce emissions from urban transport and energy production, can achieve sizeable health gains.

## Competing interests

The authors declare that they have no competing interests.

## Authors’ contributions

KN drafted the parts of the manuscript. MSH carried out the data processing, analysis of data by AirQ software and finalized the manuscript. MY carried out the some of a processing data. FM gathered the some of air quality data,population data and processed the some of data by Excell. RN drafted the parts of the manuscript. SF gathered the some of air quality data,population data and processed the some of data by Excell. AG gathered the some of air quality data,population data and processed the some of data by Excell. All authors read and approved the final manuscript.
